# An optimized LC-HRMS untargeted metabolomics workflow for multi-matrices investigations in the three-spined stickleback

**DOI:** 10.1371/journal.pone.0260354

**Published:** 2021-11-29

**Authors:** Emmanuelle Lebeau-Roche, Gaëlle Daniele, Aurélie Fildier, Cyril Turies, Odile Dedourge-Geffard, Jean-Marc Porcher, Alain Geffard, Emmanuelle Vulliet

**Affiliations:** 1 UMR-I 02 SEBIO (Stress Environnementaux et BIOsurveillance des milieux aquatiques), Université de Reims Champagne Ardenne, UFR Sciences Exactes et Naturelles, Campus Moulin de Housse, Reims cedex 2, France; 2 Univ Lyon, CNRS, Université Claude Bernard Lyon 1, Institut des Sciences Analytiques, UMR 5280, Villeurbanne, France; 3 Institut National de l’Environnement Industriel et des Risques (INERIS), UMR-I 02 SEBIO, Parc Technologique Alata, Verneuil-en-Halatte, France; Cairo University, EGYPT

## Abstract

Environmental metabolomics has become a growing research field to understand biological and biochemical perturbations of organisms in response to various abiotic or biotic stresses. It focuses on the comprehensive and systematic analysis of a biologic system’s metabolome. This allows the recognition of biochemical pathways impacted by a stressor, and the identification of some metabolites as biomarkers of potential perturbations occurring in a body. In this work, we describe the development and optimization of a complete reliable methodology based on liquid chromatography coupled to high resolution mass spectrometry (LC-HRMS) for untargeted metabolomics studies within a fish model species, the three-spined stickleback (*Gasterosteus aculeatus*). We evaluated the differences and also the complementarities between four different matrices (brain, gills, liver and whole fish) to obtain metabolome information. To this end, we optimized and compared sample preparation and the analytical method, since the type and number of metabolites detected in any matrix are closely related to these latter. For the sample preparation, a solid-liquid extraction was performed on a low quantity of whole fish, liver, brain, or gills tissues using combinations of methanol/water/heptane. Based on the numbers of features observed in LC-HRMS and on the responses of analytical standards representative of different metabolites groups (amino acids, sugars…), we discuss the influence of the nature, volume, and ratio of extraction solvents, the sample weight, and the reconstitution solvent. Moreover, the analytical conditions (LC columns, pH and additive of mobile phases and ionization modes) were also optimized so as to ensure the maximum metabolome coverages. Thus, two complementary chromatographic procedures were combined in order to cover a broader range of metabolites: a reversed phase separation (RPLC) on a C18 column followed by detection with positive ionization mode (ESI+) and a hydrophilic interaction chromatography (HILIC) on a zwitterionic column followed by detection with negative ionization mode (ESI-). This work provides information on brain, gills, liver, *vs* the whole body contribution to the stickleback metabolome. These information would help to guide ecotoxicological and biomonitoring studies.

## Introduction

Since the 1970s, it has been demonstrated that a great number of anthropogenic contaminants are present at low concentrations in the environment. They are currently defined as pseudo-persistent since they are continually released into the water bodies [1]. This environmental pressure can affect the health of the aquatic ecosystem, causing mortalities, pathological aberrations in the species development, the apparition of high resistance genes, growth retardation, increase of oxidative stress, or effect on reproductive activity [1, 2]. In aquatic ecotoxicology and biomonitoring, the three-spined stickleback (*Gasterosteus aculeatus*) has been extensively used as a worldwide sentinel fish species to investigate environmental health. Its wide distribution across Northern Europe, America and Asia (marine, brackish and fresh waters), together with its abundance and small size make it a model species easy to use and to maintain in laboratory conditions. So, for the stickleback there are many characterized biomarkers used in different contexts (passive and active) and concerning different functions [3, 4]. However, it remains difficult to have a global vision of the disturbance of the organism and to determine the links between the different individual biomarker responses.

During the last decades, broader strategies, called omics (e.g., transcriptomics, proteomics, and metabolomics) have been developed to broaden the range of potential biomarkers and to better understand contaminant mode of action, molecular target, and general ecotoxicological mechanisms [5, 6]. These approaches can provide information on early alterations from the molecular level, thus before changes in cells, tissues, or the whole organism. One of the main advantages is their ability to describe a global picture of biological processes, while individuals are unexposed or exposed. These strategies represent a promising opportunity to address the complexity of the biological processes involved in a metabolism, in order to explain the distinct signatures of various stressors [7, 8].

Among these approaches, metabolomics is a relatively recent science that involves the large-scale study of low molecular weight molecules (less than 1500 Da), namely metabolites, present in a cell, organ, or whole organism [8, 9]. Representing the most advanced level of information, they directly reflect the molecular activities of an organism. Their modulation provides direct information on altered physiological responses and signalling pathways impacted by various stressors. Some of them can then be classified as potential biomarkers of changes occurring in the organism [10]. Thus, many ecotoxicological surveys involve the use of the metabolomics approach, as it proves to be relevant for understanding the biological alterations induced by different stressors [11–13].

The choice of location, organisms, species, development stages or tissue types represent a crucial issue for the design of an ecotoxicology study. Metabolomics studies used different fish species as models such as zebrafish, fathead minnow, or rainbow trout, that allow to obtain further inputs of information. Most of them focused on whole fish homogenates [14] or muscle tissues, due to a greater ease of use or of collect, and also to have a sufficient matrix amount for analyses [15, 16]. Several authors also investigated biofluids, especially blood and bile [15, 17], again as they can be easily collected in suitable quantities. Gills and liver were also studied but to a lesser extent, mainly due to a lower amount of tissue, whereas a few studies were related to smaller tissues such as brain and kidney mainly because of inconvenience of sampling [14]. The liver represents the main compartment for the biotransformation of exogenous compounds and is also a main site for vital functions (e.g. immunity or energetic metabolism) [18, 19]. However, other tissues may react differently to stressors and could provide additional information [17, 20, 21]. They could be included in the same biological processes and then collaterally affect liver function or, on the contrary, participate in different biological pathways.

In the case of the three-spined stickleback, it is fairly difficult to collect organs since it is a small fish compared to the more often studied minnow or carp. However, organs can represent a significant source of additional information allowing to gain more insight into the metabolic mechanisms of the whole organism, in particular for a metabolomics study on a sentinel species. Hence, in this work, the study of four different matrices including whole stickleback, the liver, the brain and the gills, could provide a better understanding of this organism, exposed or not to one or several stressors.

The type and number of metabolites detected in any matrix are closely related to both the analytical method and the sample preparation used. These can be optimized in order to be adjusted to the species and the studied organs.

Thanks to the fast progress of analytical instrumentation, untargeted approaches based on high-resolution mass spectrometry (HRMS) have gained more and more popularity, achieving high sensitivity and mass resolution. Interfaced with liquid chromatography (LC), HRMS has evolved as a key analytical tool to bring out potentially a thousand endogenous compounds in various areas and matrices [13, 22], thus allowing to study the chemically complex metabolome. HRMS, used specifically with an untargeted strategy (without *a priori*), represents a valuable tool to compare metabolome information of different organs or of a whole organism as their composition, particularly with respect to lipids, can be very different. Sample preparation, LC separation, and MS detection within LC-HRMS-based untargeted metabolomics affect the nature of the molecules detected, and the quality and intensity of their detection. Historically, reversed phase liquid chromatography (RPLC) with C18 or C8 columns has been employed in untargeted LC-HRMS studies [23], due to its stability and ability to cover moderately polar to nonpolar metabolites. Nevertheless, highly polar or ionic metabolites are not effectively retained with RPLC, resulting in a loss of metabolome information. Hydrophilic interaction chromatography (HILIC) is increasingly used as complement to RPLC for water soluble metabolites analysis [24]. The combination of both RPLC and HILIC methods has already proved to be a powerful tool for metabolism analysis in several studies [25, 26]. Nonetheless, the application of HILIC for untargeted metabolic profiling is still limited, while obtaining reproducible and robust data still represents a critical issue [27, 28].

The sample preparation (extraction and enrichment/clean-up) represents a key step to achieve the full-coverage of metabolites in a minimum of steps and time, and with sufficient reproducibility. Furthermore, this preparation has to eliminate undesired matrix components, such as peptides, proteins or salts, to avoid ion suppression during the electrospray ionization process, and to maintain the separation ability of the chromatographic column. Frequently, a combination of organic solvents is used to both extract the metabolites and aggregate the proteins [24, 29]. Ultrasounds, microwave assistance, or the use of salts (salting out) have proven to be effective as a pre-treatment in biological samples [29–31]. Due to the complexity and diversity of many environmental and biological matrices, there is no universal sample preparation procedure that can be applied to all sample types.

In an ecotoxicological context, the aim of this work was to develop and optimize an untargeted LC-HRMS approach capable of covering the most exhaustive and the broadest metabolome in the whole organism and in individual organs (liver, brain, and gills) of the model species *G*. *aculeatus*. After evaluating several LC-HRMS conditions and extraction methods, we propose an analytical workflow based on a single solid-liquid extraction followed by dual HILIC(ESI-)-HRMS/RPLC(ESI+)-HRMS analyses, allowing to obtain high coverage of the metabolomes while keeping the analysis time reasonable.

## Materials and methods

### Chemicals and reagents

UPLC-MS grade acetonitrile (ACN) and methanol (MeOH) were ordered from Biosolve (Dieuze, France). Ethyl 3-aminobenzoate methanosulfonate, dimethylsulfoxyde (DMSO) (purity ≥99.7%), ammonium hydroxide (NH_4_OH) (purity ≥99%), and acetic acid (AA) (purity ≥99%) were purchased from Sigma-Aldrich (Saint-Quentin Fallavier, France). Ammonium acetate (NH_4_Ac) (purity ≥99%) and heptane (purity ≥90%) were supplied by Merck-Millipore (Saint-Quentin en Yvelines, France). Ammonium formate (NH_4_FA) (purity ≥99%) was acquired by Biosolve Chimie (Dieuze, France). Formic acid (FA) (purity 98%), isopropanol (IPA) and HPLC water were ordered by Fisher (Illkirch, France). Ammonium bicarbonate (NH_4_HCO_3_) (purity 99%) and sodium hydroxide were obtained by Fluka (Steinheim, Germany).

Labelled (^15^N, ^13^C, D) or unlabelled analytical standards (IS–S1 Table) (purity ≥97%) were purchased from Sigma-Aldrich, Fluka, CliniSciences (Nanterre, France), Chemservice (Dallas, United States), CDN isotopes (Quebec, Canada), or Merck-Millipore.

Stock solutions of individual standards at concentrations of 0.1, 0.2, or 1.0 mg/mL were prepared in ACN, H_2_O, ACN/H_2_O, DMSO, or MeOH according to their solubilities, and were stored at -18°C until use. Working standard mixtures were prepared by diluting the stock solutions with ACN.

### Three-spined stickleback maintenance, collection and preparation

Fishes were collected from the INERIS (French National Institute of Industrial Environment and Risks) husbandry, located in Verneuil-en-Halatte (France). Individuals were maintained in flow-through tanks of 600 L set at 16 ± 1°C with pH comprised between 7 and 8, at a day/night cycle of 10h/14h until use. The physico-chemical parameters of water were regularly checked. Fish were fed with frozen blood worms (3% of weight/day). Seventy-two mature males and females sticklebacks were collected at the end of the reproduction cycle in November 2018. They were then anesthetized with ethyl 3-aminobenzoate methanosulfonate to reduce stress and killed by cervical dislocation. Twenty-two animals were used for developments on whole organism and fifty were dissected to provide livers, brains and gills. They were directly frozen in liquid nitrogen and stored at -80°C until use. Whole sticklebacks or individual organs were pooled, lyophilized, and homogenized by vertical agitation (7 min at 1000 rpm) with a Genogrinder® SPEX SamplePrep® (Metuchen, USA) in polypropylene centrifuge tubes (50 mL) containing ceramic homogenizers (50 mL tubes, Agilent, France) and steel beads (3 mm, Retsch, Germany). All experiments were conducted in accordance with the Commission recommendation 2007/526/EC on revised guidelines for the accommodation and care of animals used for experimental and other scientific purposes. Furthermore, the Ethical Committee of INERIS approved all processes.

### Sample preparation

The development of the extraction was performed on pools of whole sticklebacks or organs. All assays were carried out in triplicate. For all experiments, the following overall extraction protocol was implemented: before extraction, a mass M_sample_ of sample was fortified with a volume V_mix_ of a mixture of 41 internal standards (IS–S1 Table) at a concentration C_mix_ in ACN. Both labelled and unlabelled IS were used in this mixture to represent the largest number of metabolite families during the evaluation of the various conditions. Unlabelled standards were included during these optimisation steps because they are less expensive than the labelled ones. A volume of H_2_O was added first to hydrate the matrix, then organic solvent(s) and additive(s) were supplemented. After vortexing 10 s, the extract was sonicated for 10 min and vortexed again 10 s. After centrifugation at 10,000 g during 5 min at 20°C (3K3OH, Sigma, Germany) the supernatant phases were collected. Then aliquots V_extr_ were evaporated to dryness under a slight nitrogen flow at 35°C. The dry extracts were reconstituted with a volume V_RPLC_ of ACN/H_2_O (10/90, v/v) for RPLC analysis or a volume V_HILIC_ of ACN/H_2_O (95/5, v/v) for HILIC analysis and then vortexed. The various conditions evaluated are summarized in the Table 1.

**Table 1:** Mass of sample (M_sample_), nature and volume of extraction solvent (V_solvent_), volume (V_mix_) and concentration (C_mix_) of IS mixture, extracted (V_extr_) and reconstitution volumes for RPLC (V_RPLC_) and HILIC (V_HILIC_) used during the optimization of the sample preparation steps.

Optimization step	V_solvent_ (mL)	Solvent nature	M_sample_ (mg)	V_mix_ (μg/L)	C_mix_ (mg/L)	V_extr_ (μL)	V_RPLC−_V_HILIC_ (μL)
**Nature of the extraction solvent**	7	MeOH-FA 1%/H_2_O/heptane (3/3/1,v/v/v)	50 ± 0.2 (whole fish)	500	0.5	750	75–75
	7	MeOH/H_2_O/heptane (3/3/1, v/v/v)					
	7	MeOH/CHCl_3_/H_2_O (2.5/2.5/2, v/v/v)					
**Mass sample and solvent volume**	7	MeOH/H_2_O/heptane (3/3/1, v/v/v)	50 ± 0.2 and 25 ± 0.2 (whole fish, liver, brain, gills)	105	3.5	750 or 245	100–100
	2.5	MeOH/H_2_O/heptane (1.1/1.1/0.3, v/v/v)					
**Solvent extraction ratio**	2.5	MeOH/H_2_O/heptane (1.1/1.1/0.3, v/v/v)	50 ± 0.2 whole fish, liver, brain	150	3.5	245	100–100
	2.5	MeOH/H_2_O/heptane (1.7/0.5/0.3, v/v/v)		150	3.5	245	100–100
	2.5	MeOH/H_2_O/heptane (0.5/1.7/0.3, v/v/v)	25 ± 0.2 gills	150	3.5	245	100–100
	2.5	MeOH/H_2_O/heptane (0.83/0.83/0.83/, v/v/v)		150	3.5	245	100–100

The final extraction process was as followed (S1 Fig): aliquots of M_sample_ = 50 mg (± 0.2 mg) of whole fish, liver, or brain, and 25 mg (± 0.2 mg) of gills were weighed in a 5 mL tube. A solid-liquid extraction was performed with 850 μL of water, 250 μL of MeOH, and 150 μL of heptane (whole fish), with 250 μL of water, 850 μL of MeOH, and 150 μL of heptane (liver and brain), or with 550 μL of water, 550 μL of MeOH, and 150 μL of heptane (gills). After vortexing 10 s, the extract was sonicated for 10 min and vortexed again 10 s. Next, samples were centrifuged at 10,000 g for 5 min at 20°C. For all matrices, the extraction step was repeated once to enhance the extraction efficiency. After the first extraction, 740 μL of supernatant were transferred into a 5 mL tube. After the second extraction, 1 mL was transferred into the same tube. Finally, two aliquots (V_extr_ = 245 μL) of this extract were transferred into two 2 mL tubes for RPLC and HILIC analyses, respectively. The aliquots were then evaporated to dryness under a slight nitrogen flow at 35°C. The dry extracts were reconstituted in V_RPLC_ = 100 μL of ACN/H_2_O (10/90, v/v) and V_HILIC_ = 100 μL of ACN/H_2_O (95/5, v/v).

### Liquid chromatography-QToF-mass spectrometry

LC-HRMS analyses were achieved with an Ultimate 3000 UHPLC system (Thermo Scientific®, MA, USA) coupled to mass spectrometry using a quadrupole time-of-flight mass spectrometer (QToF) (Maxis Plus, Bruker Daltonics®, Bremen, Germany). The QToF system was equipped with an electrospray ionization interface (ESI) setting in positive or negative modes. Compounds were separated using an Acquity UPLC® HSS T3 C18 (100 x 2.1 mm ID, 1.8 μm) column from Waters (Saint-Quentin en Yvelines, France) or a Nucleodur HILIC (100 x 2 mm ID, 3 μm) column from Macherey-Nagel (Hoerdt, France) both equipped with a UHPLC in-line filter (KrudKatcher, Phenomenex®) to prevent columns from prematurely clogging.

The final optimized mobile phases for RPLC were composed of (A) H_2_O with 0.01% FA and (B) MeOH and the elution gradient was as followed: 0–1 min, 1% MeOH; 1–15 min, from 1% to 100% MeOH; 15–19 min, 100% MeOH and from 19 to 23 min back to 1% MeOH. The flow-rate was set at 0.3 mL/min. The compounds were then detected in the positive ionization mode (ESI+). For HILIC, the optimized mobile phases were composed of (A) 2 mM NH_4_Ac + 2 mM NH_4_OH in H_2_O pH 8.0 and (B) ACN. The elution gradient was as followed: 0–2 min, 95% ACN; 2–18 min, from 95% to 50% ACN; 18–23 min, 50% ACN and from 23 to 33 min, back to 95% ACN. The flow rate was set at 0.4 mL/min. The compounds were then detected in the negative ionization mode (ESI-). The oven temperature was maintained at 40°C for both columns and the sample injection volume was fixed at 5 μL.

Mass spectra were acquired in full-MS scan mode at a resolution of 20.738 (FWHM) at m/z = 376.0381 over the m/z range [50–1000 Da] for (ESI+) and [80–1200 Da] for (ESI-), with a scan rate of 1 Hz. ESI source parameters were set as followed: electrospray voltage 3.6 kV in ESI+ and 3.5 kV in ESI-, nebulizer pressure 3 bar (N_2_), and drying gas flow rate 9 L/min (N_2_). An external calibration of exact masses was systematically performed at the beginning of each run, using a solution of sodium formate and acetate (0.5 mL of 1 M NaOH + 25 μL FA + 75 μL AA in in H_2_0/IPA 50/50 v/v), generating cluster ions [M+H]^+^ in the range 90.9766–948.8727 Da with high precision calibration (HPC) mode at a search range ± 0.05 m/z. Accepted standard deviations were inferior to 0.5 ppm.

The softwares OTOF Control 4.1 and HyStar^TM^ 4.1 (Bruker Daltonics®) were used to acquire and process data. After HRMS acquisition, the data were processed via Compass DataAnalysis 4.3 and Metaboscape 4.0 softwares from Bruker Daltonics®. The extracted ion chromatograms (EIC) correlation value was 0.9, the minimum peak length was 8 spectra and the retention time (R_T_) lapse was [0.5–20 min]. The data pre-processing including peak detection or peak picking, peak grouping, and peak alignment was directly performed on Metaboscape. The peak picking corresponds to the detection of a number of points allowing to modelize the peak shape. The peak alignment consists of recognizing peaks with the same m/z and R_T_ in the different datasets and to associate these features under a single entity throughout the samples. Peak grouping batched the corresponding adducts, ions clusters (dimere, trimere…), fragments, isotopes related to a same molecule. Generation of EIC for the adducts [M+H]^+^, [M+Na]^+^, [M+NH_4_]^+^ in ESI+ or [M-H]^-^, [M-H_2_O-H]^-^ in ESI- was performed to detect the 41 IS (S1 Table). The integration of the EIC and retrieval of an average spectrum across the peak were then processed to compare and choose the adequate separation and sample preparation conditions.

## Results and discussion

### Matrices of interest

In the present study, we considered both the whole fish, in order to get a broad response from the organism, but also three individual organs: liver, brain, and gills. Liver was considered because of its key role in fish metabolism. Indeed, it constitutes a global view of the effects of various stressors on the biological signaling pathways. It is the major biotransformation and detoxification organ helping to eliminate exogenous compounds. It also plays a main role for vital functions regarding different biological processes such as insulin-like growth factor, energetic metabolism, or production of hormones. As a consequence, it is an important toxicological target location for a great number of contaminants such as pharmaceuticals or metals [15, 32].

Gills were considered since they are the respiratory organs of fish and one of the main sites in interaction with the environment [21]. Their major role is to maintain the respiratory function by assimilation of oxygen from the water. They also play an excretion role helping the kidney to remove waste products, especially the ammonia [33, 34]. Moreover, they are involved in the osmoregulation (regulation of minerals) and in the maintaining of a constant pH in the body [35].

Finally, to the best of our knowledge, among all fish tissues, the brain represents the least studied one [14] partly due to its poor understanding and its very low amount. Thus, little information is available on its functioning and its signaling pathways. Nonetheless, some studies demonstrated that fish brain can be a target organ for some pollutants, especially pesticides [21, 32]. Thus, it was also considered in this metabolomics development since it is the nervous organ and it is fragile to toxicant. The development of a suitable analytical method would be useful to further evaluate its interest for metabolomics or environmental research.

### Optimization of LC-HRMS conditions

First of all, the optimization of the method consisted in selecting the best separation and detection conditions. Eighteen labelled IS representative of various intracellular metabolites with contrasting physico-chemical properties (amino acid and derivative, sugar, lipid, organic acid, nucleoside, peptide, vitamin, hormone, fatty acid) were first selected to optimize LC conditions. Matrix effects are a widely known and observed phenomenon during electrospray ionization of complex matrices. To meet the conditions of matrix effects, the optimization tests were performed in fish extracts spiked with a mixture of IS. This extract was obtained after a simple solid-liquid extraction of 50 mg of whole sticklebacks using 13 mL of a ACN/heptane mixture (10/3, v/v).

In order to cover a wide range of metabolites, we chose to study the two orthogonal chromatographic modes RPLC and HILIC. For RPLC mode, a universal C18-bonded silica column (spherical particles) was selected for the separation of the least polar to moderately polar compounds. This column is compatible with 100% aqueous mobile phase and the operating range for this column is 2 to 8. For HILIC, a zwitterionic column (ZIC-pHILIC) containing spherical silica modified with both ammonium and sulfonic ligands was chosen for the separation of the ionic and most polar compounds. The operating pH range of this column is 2 to 8.5.

The main reason for not detecting a metabolite during untargeted screening is because of its inability to ionise under the conditions applied. This is partly conditioned by the pH value of the mobile phase and the pKa of the metabolite. Another possible reason is the existence of matrix effects in complex matrices like fish extracts, that can promote suppression of the signal during electrospray processing. It is therefore very difficult and risky to predict the best conditions for the analysis.

To optimize chromatography conditions coupled to ESI-HRMS, we applied ten chromatographic conditions (Table 2) to investigate the influence of the solvent nature, of the mobile phase’s pH, and of the salt additive nature. We considered both the ionization efficiency of the IS detected, the numbers of features detected in this complex matrix (after subtraction of the analytical blank), and the reproducibility of the analyses (R_T_ and intensity of the signals). Minimal R_T_ and peak shape variations are also crucial for untargeted metabolomics analysis, in order to ensure alignment and subsequent statistical analysis. These parameters were also taken into account to select the best compromise in the chromatographic conditions.

**Table 2:** Mobile phase conditions evaluated with the C18 and ZIC-pHILIC columns.

Column and ESI mode	Aqueous phase (A)	Organic phase (B)
C18 / ESI+ and ESI-	0.01% FA in H_2_O pH 4.0	ACN
C18 / ESI+ and ESI-	0.01% FA in H_2_O pH 4.0	MeOH
ZIC-pHILIC / ESI+ and ESI-	5mM NH_4_FA + 0.05% (v/v) FA in H_2_O pH 2.2	ACN
ZIC-pHILIC / ESI+ and ESI-	5mM NH_4_FA + 0.01% (v/v) FA in H_2_O pH 4.8	5mM NH_4_FA + 0.01% (v/v) FA in ACN/H_2_O (90/10)
ZIC-pHILIC / ESI+ and ESI-	5mM NH_4_Ac + 0.025% (v/v) AA in H_2_O pH 4.8	ACN
ZIC-pHILIC / ESI+ and ESI-	10mM NH_4_HCO_3_ in H_2_O /ACN (90/10) pH 8.2	ACN
ZIC-pHILIC / ESI+ and ESI-	10mM NH_4_Ac + 10mM NH_4_OH in H_2_O pH 9.2	ACN
ZIC-pHILIC / ESI+ and ESI-	5mM NH_4_Ac + 5mM NH_4_OH in H_2_O pH 8.5	ACN
ZIC-pHILIC / ESI-	2mM NH_4_Ac + 2mM NH_4_OH in H_2_O pH 8.0	ACN
ZIC-pHILIC / ESI-	1mM NH_4_Ac + 1mM NH_4_OH in H_2_O pH 7.8	ACN

FA: formic acid; MeOH: methanol; ACN: acetonitrile; NH_4_FA: ammonium formate; NH_4_Ac: ammonium acetate; AA: acetic acid; NH_4_HCO_3_: ammonium bicarbonate; NH_4_OH: ammonium hydroxide.

Gradient program for RPLC conditions: 1% B for 1 min then increase to 100% B within 14 min; flow-rate 0.3 mL/min.

Gradient program for HILIC conditions: 95% B for 2 min then decrease to 50% B within 16 min; flow-rate 0.4 mL/min.

#### Optimization of RPLC conditions

**Fig 1:**
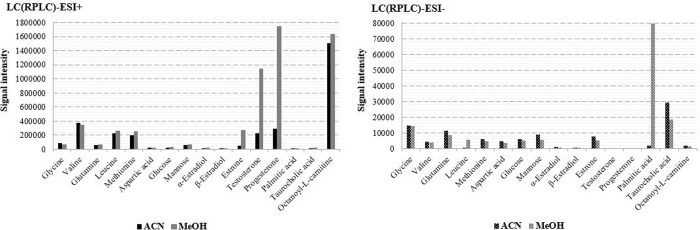
Intensities of targeted metabolites analyzed in LC(RPLC)-(ESI+/-)-HRMS conditions with MeOH or ACN as organic mobile phase B, after extraction of 50 mg of fish spiked at 500 μg/L.

As they can affect ionization, and thus detection efficiency as well as background signal, we studied two organic mobile phases, ACN and MeOH, with both ionization modes. Our results showed that with ESI+, the use of MeOH instead of ACN had a positive impact on the ionization intensity of most IS compounds (Fig 1). Indeed, ionization was increased for 10 targeted compounds over 16 analyzed on the C18 column, and slightly decreased for the amino acids valine, glycine and aspartic acid. The increase was especially noticeable in the case of hormones, with intensities enhanced by a factor 5 to 8 with MeOH. On the contrary, with ESI- most compounds presented higher intensities with ACN, except palmitic acid that exhibited a very low intensity and leucine which was no longer detectable with ACN. When considering the compounds detected in both positive and negative modes, 12 of the 14 corresponding compounds showed higher intensities in the positive mode, from 4 to 1000 times higher using MeOH and from 2 to 800 times higher with ACN as organic modifier.

Moreover, the total number of features was nearly twice higher with MeOH than ACN (4541 *vs* 2408) when the analysis was conducted in the positive ionization mode. The difference was lower in the negative mode (1046 *vs* 875).

Consequently, MeOH was chosen as organic mobile phase since it resulted in higher target metabolites intensities and in a greater number of features.

#### Optimization of HILIC conditions

For the HILIC conditions, the impact of different additives (NH_4_FA, NH_4_Ac, NH_4_HCO_3_, NH_4_OH, FA, AA) in the mobile phases was studied (Table 2). Indeed, the presence of buffers or acid additives has a significant impact on the ionization efficiency. It also determines mobile phase pH and regulate stationary phase selectivity, and can have a strong impact on peak shape.

**Fig 2:**
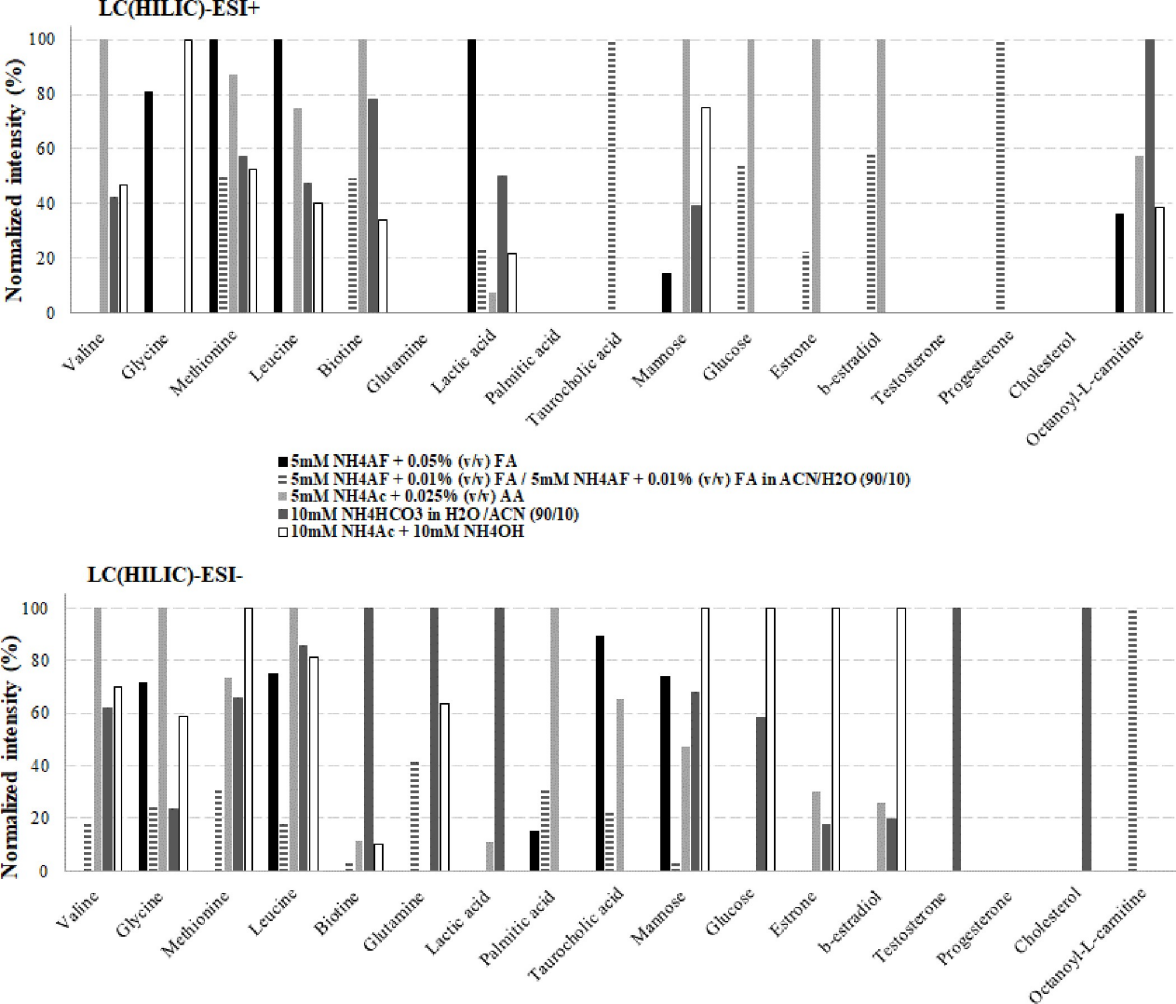
Normalized intensities of targeted metabolites analyzed by LC(HILIC)-(ESI+/-)-HRMS using different mobile phase conditions (results obtained after extraction of 50 mg of fish spiked at 500 μg/L).

The mobile phase buffer concentration as well as the presence of additives in only one or both mobile phases were also considered in our study, since they can have a strong impact on peak shape in HILIC. The gradient program was kept unchanged for all conditions. The detailed tested conditions are summarized in Table 2. The comparison was based on both the compounds intensities from fish extract spiked with IS mixture, and the total number of detected features. The normalized intensities of the IS detected in HILIC mode with positive and negative ionization modes are shown in Fig 2.

*Addition of salts in aqueous and/or organic mobile phases*. In order to guarantee better reproducibility and peak shape in HILIC, it is often recommended to maintain a constant ionic strength throughout the chromatographic run, and therefore to use the same amount of salts in both the aqueous and organic phases. So we first compared the data obtained using NH_4_FA and FA either only in the aqueous mobile phase, or in both aqueous and organic mobile phases. We observed that the presence of salts in both phases promoted the ionization of several of the targeted metabolites (such as palmitic acid, methionine, glutamine, or biotin) but decreased the intensity of others (such as glycine, leucine, and mannose) in negative ionization mode. For ESI+, it decreased the intensity of glycine, methionine, lactic acid, or mannose while increasing the signals of biotin, taurocholic acid, or hormones. The total number of detected IS was higher using salts in both phases, but the overall number of detected features was lower by a factor 2 when both mobile phases contained salts (1150 *vs* 2570). Regarding the retention, the presence of salts in both the aqueous and organic phases induced a decrease in retention (from 4 to 30%, i.e. from 1 to 7 min) for whole compounds detected with both conditions, except palmitic acid whose R_T_ was not affected. Moreover, no difference in peak shape was noticed between both HILIC conditions.

Thus, our results showed that for these conditions, it is better to add salts in the aqueous mobile phase only.

*Influence of additive nature*. We compared the data obtained using NH_4_FA or NH_4_Ac as additive. For the same pH value (4.8) and a roughly comparable ionic strength, the use of NH_4_Ac instead of NH_4_FA generally favored the signal intensity of the IS by a factor of 1 to 5 in ESI- (except for glutamine, testosterone, and octanoyl-L-carnitine whose signals were no longer detectable with a correct peak shape) (Fig 2). As an example, the mannose signal was increased 15 fold in ESI- and by a factor of 100 in ESI+ with the use of NH_4_Ac compared to NH_4_FA. The number of target metabolites detected in ESI+ was also higher with NH_4_Ac (11 *vs* 9) as well as the total number of features (40% higher) observed in both ionization modes. Regarding the retention, the use of NH_4_Ac instead of NH_4_FA increased the retention (from 4 to 22%, i.e. from 1 to 5 min) for the compounds detected in both conditions, except palmitic acid whose R_T_ was not impacted. Moreover, the use of NH_4_Ac allowed to obtain a better peak shape (number of points per peak) for 5 IS compared to NH_4_FA.

*Influence of mobile phase pH and salt concentration*. We evaluated the impact of a more acidified mobile phase (pH = 2.2) composed of NH_4_FA and FA. It allowed the detection of only 6 IS in the positive mode, and 5 in the negative one. At pH 4.8 with the same salts nature, a higher number of compounds was detected, 8 and 10 IS, in the positive and negative modes, respectively, especially amino acids.

On the one hand, quite intuitively, acidic conditions (pH = 2.2 and 4.8) favored the positive ionization mode of the targeted compounds. On the other hand, the total number of features was always lower during positive ionization mode than in the negative one (by a factor 2 to 5).

When considering basic conditions, the use of the bicarbonate mobile phase promoted, in ESI- mode, the ionization of biotin, glutamine, lactic acid, and testosterone. With ESI+, it showed a significant interest only for octanoyl-L-carnitine. It can also be noted that the use of NH_4_HCO_3_ was the only condition that allowed a low detection of cholesterol, although not retained on the column. In terms of total number of features, with this mobile phase 780 features were detected in positive mode and 1490 in negative mode.

The use of NH_4_OH in combination with NH_4_Ac in the negative mode led to the detection of 10 IS including amino acids, vitamin, sugars, and hormone families. Moreover, among the detected IS, 5 had the highest intensities between whole tests. The total number of features obtained with ESI- was 1515.

So, with the evaluation of the impact of different additives in the mobile phases, we concluded that the maximum number of detected IS exhibiting a good peak shape and highest IS intensities were obtained under conditions involving the highest salt concentrations, and corresponding to basic pH (10 mM NH_4_HCO_3_, pH 8.2 and 10 mM NH_4_Ac + 10 mM NH_4_OH, pH 9.2).

*Clogging of the mass spectrometer source due to salts use*. We observed that the use of NH_4_HCO_3_ or NH_4_Ac + NH_4_OH induced clogging in the system.

This effect was so important with NH_4_HCO_3_ that it was not added for further tests. Indeed, after visual examination of the electrospray probe and conical shield, salt deposition appeared after a single sequence of 14 injections (about 8h). Although they may be of high interest for occasional analyses, they cannot be considered for metabolomics purposes requiring high throughput analyses with great reproducibility in terms of signal intensity.

**Fig 3:**
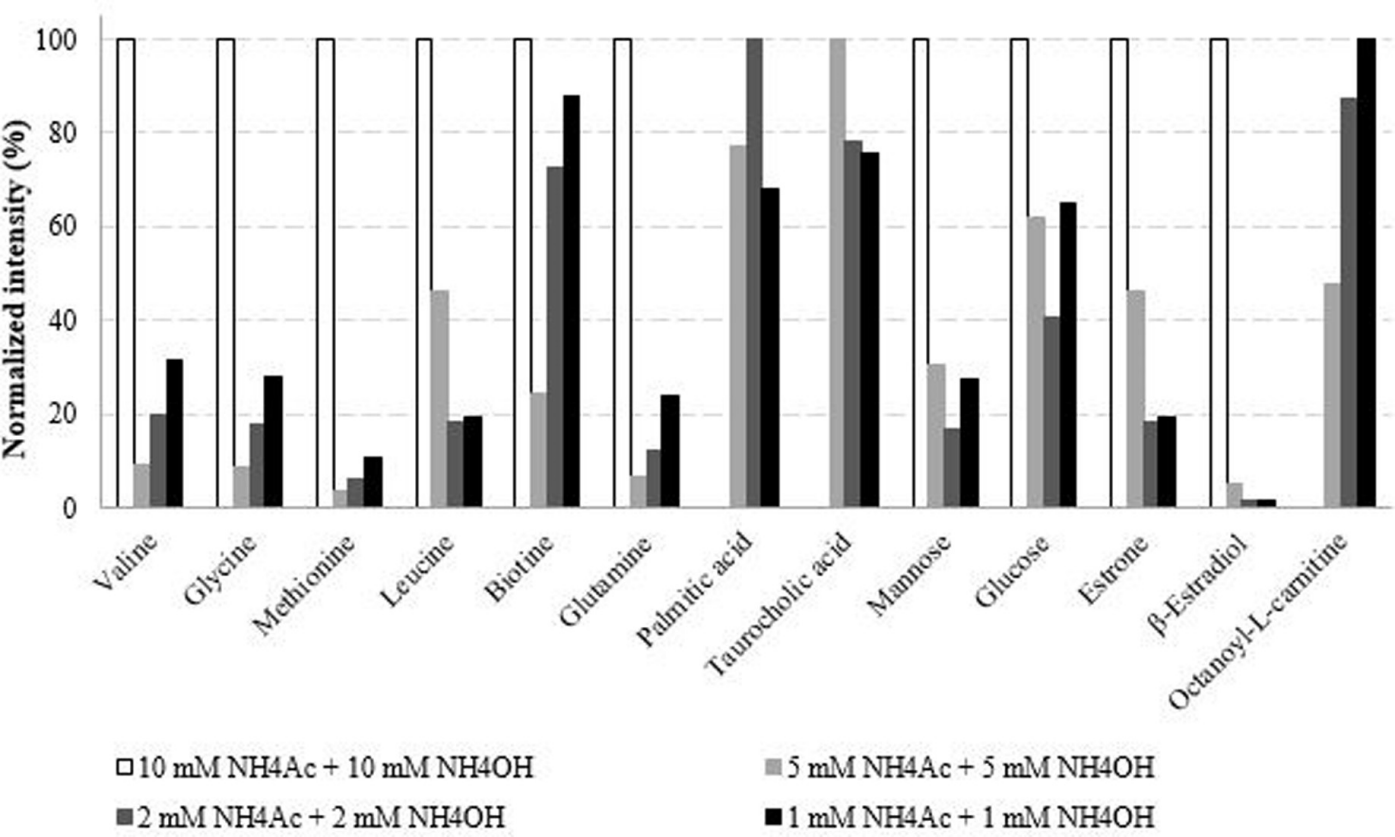
Normalized intensities of targeted metabolites analyzed by LC(HILIC)-(ESI-)-HRMS using various concentrations of (NH_4_F + NH_4_OH) salts in the mobile phase (results obtained after extraction of 50 mg of fish spiked at 500 μg/L).

In order to ensure reproducible metabolomic analyses over the long term, and to preserve the mass spectrometer, we progressively decreased NH_4_Ac + NH_4_OH salt concentrations (Fig 3). This had an important impact on the nature of the compounds detected, their number, and the intensity of the signals. Palmitic acid and taurocholic acid were no more detected with the highest salt concentration (10 mM), but were detected at lower concentrations. The high proportion of salts can result in ionization suppression. The total number of features detected in negative ionization mode was 1515, 2616, 1991, and 2635 for salt contents of 10 mM, 5 mM, 2 mM, and 1 mM, respectively. We observed that the evolution of the ionic strength with the same nature of salts did not show a significant difference in the IS R_T_. The same R_T_ reproducibility was obtained with 5, 2, and 1 mM of salts (deviation of 0–0.4%). Nevertheless, fewer IS were time deviated with a salt content of 2 mM (one *vs* four with 5 and 1 mM).

Consequently, the mobile phase with 2 mM of ammonium salts was considered as the best compromise since it resulted in suitable IS intensities, a proper number of features, and a good R_T_ reproducibility. Ivanisevic *et al*. [25] previously studied different mixtures of NH_4_Ac and NH_4_OH for the analysis of hydrophilic and central carbon metabolites with an aminopropyl-based HILIC column. Their optimal conditions were close to ours since they finally chose a composition of 10 mM of each salt with a low flow-rate of 0.05 mL/min.

*Evaluation of column reequilibration time*. Finally, we also investigated the column reequilibration time that represents a critical parameter in HILIC methods. Insufficient column equilibration may cause shifting of the R_T_, resulting in poor reproducibility. The evaluation of the reequilibration time was performed with triplicate injections of a mixture of IS in ACN/H_2_O (95/5; v/v). We compared the R_T_ deviation of each target compound between the first and the third injections. Different equilibrium delays between two injections were evaluated namely 10, 20, 25, and 30 minutes, which corresponds to approximately 13, 26, 32, and 38 column volumes, respectively. Not intuitively, the lowest drift times were observed with 10 minutes. The deviation was inferior to 0.4% for whole IS (i.e. drift time inferior to 0.1 min). Time shifts over the ranges (0.4% - 0.9%), (0.4% - 1.7%), and (0.9% - 5.7%) were observed after an equilibration time of 20, 25, and 30 minutes, respectively. A relatively fast delay between each analytical run was therefore sufficient to achieve good repeatability. We thus applied a 10-minutes equilibration time at the beginning of each analysis. In order to confirm this reproducibility over a longer injection sequence, a series of 30 successive injections (corresponding to a 15h sequence) was performed. It still showed R_T_ deviations comprised between 0 and 0.4% for whole IS.

Over the past few years, some works have put in evidence that, if full re-equilibration of HILIC phases can take a long time (tens of minutes or hours), highly repeatable HILIC separations can be obtained rapidly. During his investigation on full and partial equilibration of HILIC phase, McCalley highlighted a repeatable equilibrium achieved after only 4.3 min [36]. The conclusions of Stoll and Seidl [37] were consistent with the obtention of high repeatable HILIC separations with very short re-equilibration times.

#### Conclusion on LC-HRMS conditions

An ideal experiment would analyse the samples on four platforms: hydrophilic compounds on both RPLC and HILIC columns and more hydrophobic compounds on a RPLC column, both with detection in positive and negative ionization modes. According to the number of IS detected, their intensities and the number of features, the combination of two injections, one on RPLC with ESI+ (A: 0.01% FA in H_2_O; B: 100% MeOH) and one on HILIC with ESI- (A: 2 mM NH_4_Ac + 2 mM NH_4_OH in H_2_O; B: ACN) was chosen instead of four injections, in order to save time, cost, and quantity of sample. This strategy was therefore considered to largely cover fish metabolome.

It is worth noting that the final RPLC and HILIC conditions allowed separating the amino acids isomers leucine and isoleucine. However, none of the LC conditions investigated allowed separating the couples of sugar isomers, namely fucose/rhamnose (RPLC R_T_ = 1.0 min), fructose/galactose (RPLC R_T_ = 0.8 min, HILIC R_T_ = 2.4 min) and maltose/saccharose (RPLC R_T_ = 0.9 min, HILIC R_T_ = 6.3 min).

### Development of the sample preparation

The LC-HRMS conditions established, we then optimized the sample preparation. Among the sample preparation techniques, we chose solid-liquid extraction, which optimization allowed both the extraction of a wide variety of metabolites and the precipitation of proteins [29]. An ultrasound step was systematically added to induce a greater solvent penetration into the matrix, leading to an enhancement of metabolite extraction efficiency.

In order to choose the best compromise for the extraction conditions, we considered not only the response of the internal standards initially added to each sample, but also the total number of features, as well as the repeatability of the signals. We did not calculate the extraction yields of the analytical standards, but considered their signal intensities. Indeed, these are taking into account both extraction efficiency and matrix effects. Several parameters were independently studied: extraction solvent composition (nature, volume, and ratio) and initial sample mass.

#### Nature of the extraction solvents

The nature of the extraction solvents is unquestionably the major factor affecting the nature, number, and abundance of metabolites detected in biological samples. Given the great physico-chemical variety of metabolites to be extracted in untargeted investigations, a solvent mixture has to be considered. Our strategy was based on a ternary combination of hydrophilic, lipophilic and medium-polarity solvents. In environmental metabolomics studies, H_2_O with MeOH or ACN, and/or a lipophilic solvent such as CHCl_3_, MTBE or hexane were commonly used [30, 38]. Here, we considered combinations of solvents with MeOH / H_2_O / CHCl_3_ or heptane (Table 1). These combinations led to the formation of two phases. The resulting disadvantage is the possible fractionation of the semi-polar compounds in variable proportions between the hydrophilic and lipophilic phases. In order to avoid losing compounds and to cover the whole metabolome, we did not analyse separately the polar fraction with HILIC conditions and the less polar one with RPLC conditions. We pooled the fractions, then evaporated and reconstituted them in solvents compatible with RPLC and HILIC analyses, respectively. Thus, the same extract was analyzed both with RPLC and HILIC optimized conditions.

Moreover, we evaluated the effect of adding formic acid to the extraction solvent, as the addition of acid in low proportions could protonate some metabolites, improving their solubility and thus the efficiency of their extraction [29].

**Fig 4:**
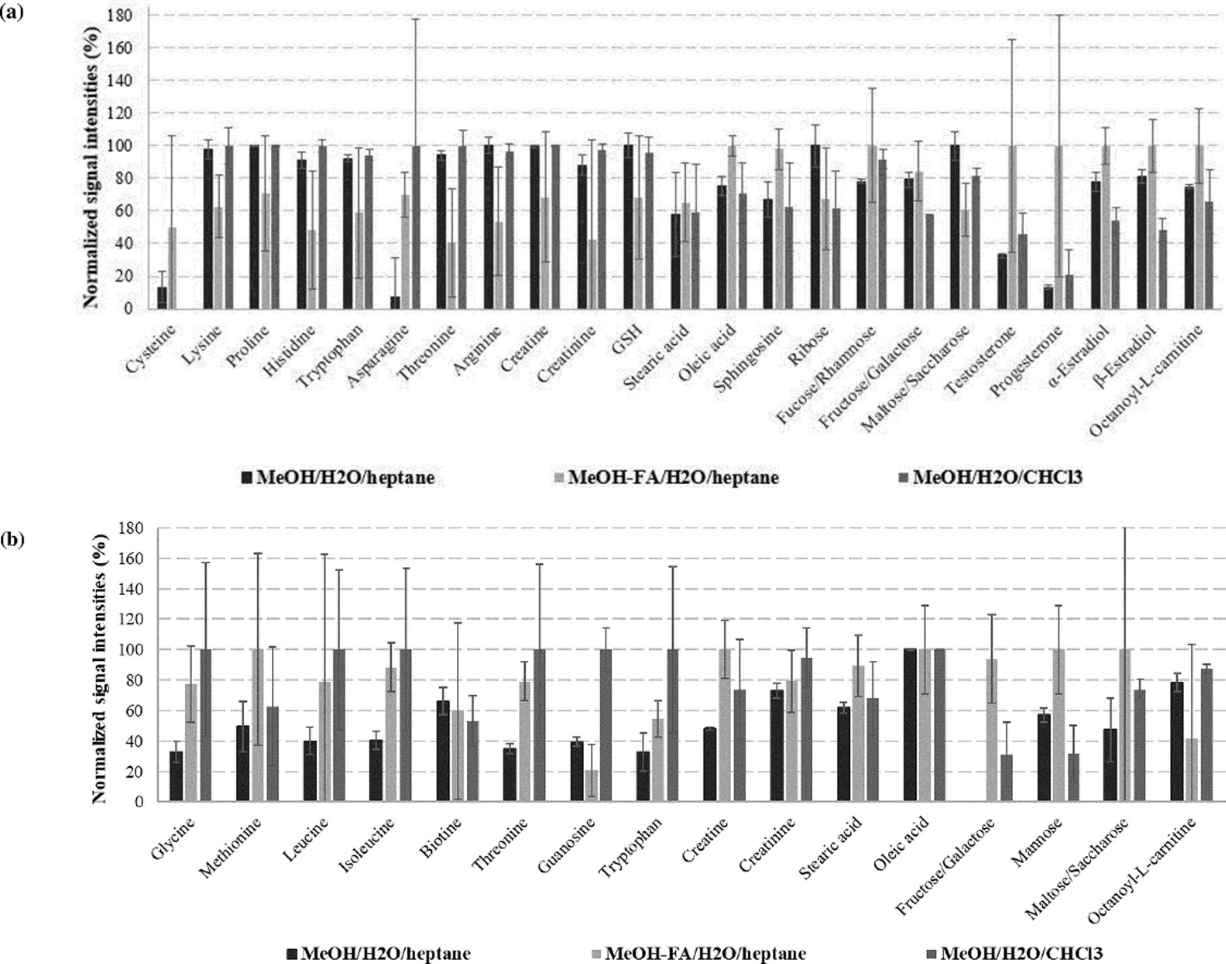
Normalized intensities of internal metabolites analyzed by (a) RPLC(ESI+) and (b) HILIC(ESI-) after extraction (n = 3) with various solvent mixtures (extraction of 50 mg of fish spiked at 500 μg/L).

We first studied the normalized intensities of the LC-HRMS responses of the internal standards after triplicate extractions of 50 mg of fish with different solvent mixtures (Fig 4A and 4B).

The incorporation of formic acid increased the intensities of the amino acids analyzed with HILIC(ESI-) conditions and slightly decreased the intensities of those analyzed with RPLC(ESI+) conditions. In both cases, the repeatability was strongly degraded in the presence of acid.

The nature of the hydrophobic solvent showed a strong impact on both amino acids signal and repeatability. Indeed, a significant increase in signal intensity was observed when using CHCl_3_ in the extraction, during the HILIC(ESI-) analysis. However, the extraction was not repeatable (relative standard deviation–RSD–values higher than 30%). The use of heptane therefore allowed getting both high LC-HRMS responses and good repeatability for amino acids.

The signal intensities of sugars were only slightly affected by the choice of the hydrophobic solvent. On the other hand, the addition of acid strongly decreased the repeatability of their extraction.

It is worth noting that for the most hydrophobic compounds (fatty acids, sphingolipid, hormones), the use of heptane or CHCl_3_ had a low impact on average signal intensity or repeatability, whereas the addition of formic acid degraded their repeatability.

It is interesting to note that for the metabolites detected in both RPLC and HILIC conditions, normalized peak intensities vary between RPLC(ESI+) and HILIC(ESI-) even though the same extracts were injected (except reconstitution solvents). This is due to matrix effects. The different molecules that elute and ionise together with the analytes in the electrospray source cause their inhibition or signal enhancement. These effects are very different between ESI+ and ESI- for two reasons. Firstly, it is not the same molecules that elute simultaneously with the analytes in RPLC and HILIC. Secondly, the ionisation conditions (positive or negative mode, different solvents and desolvated salts) are different between the two types of analysis.

We then evaluated the total number of detected features according to the conditions. An average of 3161 ± 308 were recorded for the RPLC(ESI+) conditions; acidified conditions did not affect the number of features.

On the contrary, under HILIC(ESI-) conditions, the number of features was affected by the presence of acid, as the number decreased from 3265 ± 504 (without acid) to 1356 ± 40 (with acidified MeOH).

Considering these results, the mixture MeOH/H_2_O/heptane was the best option to obtain the highest signal intensities of the standards with a good repeatability, together with the highest number of features.

#### Sample mass and volume of the extraction solvents

The sample mass is responsible for the quantity of detected metabolites but it is also involved in the matrix effects generated during the electrospray ionization. Similarly, the total volume of solvent is important to evaluate, in order to preconcentrate as much metabolites as possible while limiting signal losses due to matrix effects. Adequate ratio sample/solvent volume is also involved in the suitable dispersion and homogenization between the matrix and the solvent.

Thus, extractions of 25 or 50 mg of whole fish, liver, gills, or brain, performed with 2.5 or 7 mL of the MeOH/H_2_O/heptane mixture (3/3/1, v/v/v) were evaluated (Table 1). According to these extraction mixture volumes, aliquots of 245 μL or 750 μL of each extract were transferred for RPLC and HILIC analyses, respectively.

We first studied the differences in signal intensity and repeatability when 50 mg of whole fish, liver, brain, and gills were extracted with 2.5 mL or 7 mL of solvent (S2–S5 Figs). With RPLC(ESI+) analysis, the mean intensities were generally higher after extraction with 2.5 mL of solvent. The signal repeatability was mostly lower with 2.5 mL than with 7 mL in fish extracts, even up to 5-fold lower for the hormones (with RSD 6–13.5% and 27.5–42.5% for 7 mL and 2.5 mL, respectively). On the other hand, the signal repeatability was the same with 2.5 mL or 7 mL for liver, brain, and gills extracts (with a mean of 13% for both volumes). With HILIC(ESI-) conditions, mean intensities for the four matrices were also higher using 2.5 mL rather than 7 mL. The change in the mass/solvent ratio had globally a low impact on signal repeatability in fish, liver, or gills extracts. On the other hand, it improved the signal repeatability in brain extracts (mean RSD of 23% with 7 mL and 12% with 2.5 mL).

When the initial mass of sample was reduced to 25 mg, while maintaining a solvent volume of 2.5 mL, the effects observed were not the same according to the matrix. Thus, for fish, it was difficult to identify a common trend among all the internal standards used, or even within the same family. For example, the intensity of leucine doubled for a mass of 50 mg compared to 25 mg, while the signal of another amino acid, guanosine, decreased by one-third. For liver, the mean intensities were higher after extraction of 50 mg than 25 mg. It was the opposite in the case of gills. For brain extracts, the mean intensities were equivalent after extraction of 25 mg and 50 mg. With respect to repeatability, it was broadly equivalent whether considering an initial mass of 50 mg or 25 mg, except for the liver for which repeatability was improved with 50 mg (mean RSD of 3.7% with 50 mg and 10.1% with 25 mg).

**Fig 5:**
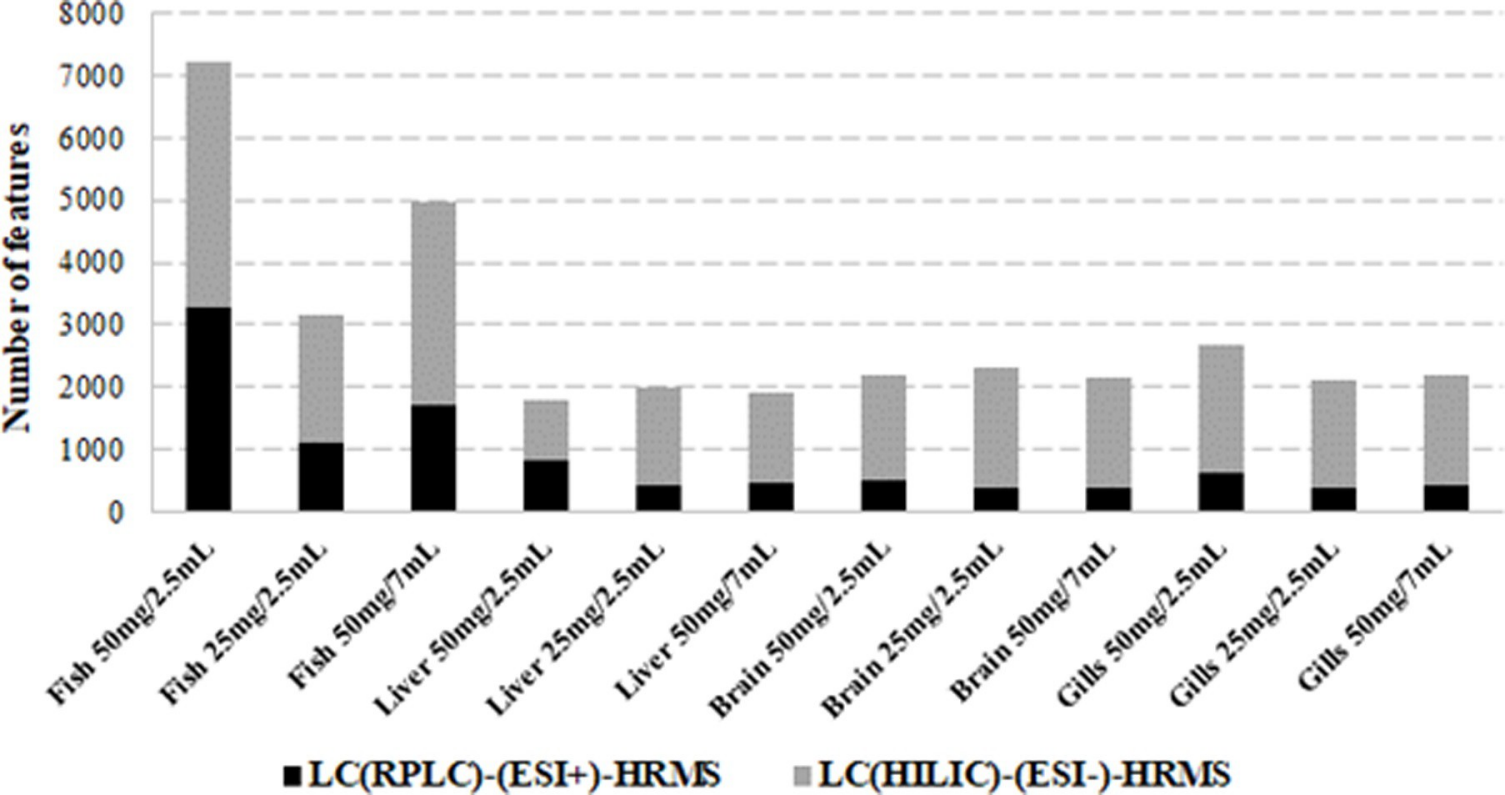
Number of features (m/z, R_T_) detected with different sample mass and volume of the extraction solvents in fish, liver, brain or gills, analyzed in LC(RPLC)-(ESI+) and LC(HILIC)-(ESI-) HRMS.

We then evaluated the number of features detected, for each organ and each extraction condition (Fig 5). First, as expected, the total number of features detected was lower in the individual organs compared to the whole body (between 2.7 and 4 times lower depending on the organ and extraction condition). It is also noticeable that the number of features was higher with HILIC(ESI-) conditions than RPLC(ESI+) conditions (up to 5 times more).

A positive influence of the decrease in extraction solvent volume (from 7 mL to 2.5 mL) on the number of features detected in whole fish was observed in both RPLC(ESI+) and HILIC(ESI-) conditions. The number of compounds increased by a factor of 3.8 when using 2.5 mL. In the same way, increasing the initial sample mass allowed the detection of a greater number of signals in the whole fish. The number of features increased by a factor 2.3 when increasing the matrix amount from 25 mg to 50 mg. The effect of solvent volume or initial sample mass was less pronounced for individual organs, although the number of substances with RPLC(ESI+) conditions increased slightly with decreasing extraction volume or increasing initial mass. It can be hypothesized that matrix effects were also greater and therefore partially compensated by the increase of metabolites present in the extracts.

Considering these results, best extractions were obtained using 50 mg of whole fish, liver, or brain and 25 mg of gills with 2.5 mL of the solvent mixture MeOH/H_2_O/heptane. It is worth noting that theses masses make it possible to work at the scale of one individual for the whole organism, the liver and the gills. On the other hand, it is necessary to pool two or three fish brains to get a sufficient amount of matrix.

#### Extraction solvents ratio

In order to determine the best solvent ratios, four proportions of MeOH/H_2_O/heptane, namely (1.1/1.1/0.3; v/v/v), (0.5/1.7/0.3; v/v/v), (1.7/0.5/0.3; v/v/v), (0.83/0.83/0.83; v/v/v) were finally compared (S6–S9 Figs).

*Whole fish*. In the case of whole fish, the maximum intensities were mostly obtained with the two compositions containing the highest proportions of water. Intensities were especially higher for the sugars ribose, fructose, galactose, saccharose, and maltose. The mean RSD for intensity was 9% (RPLC condition) and 10% (HILIC condition) with the condition including more water.

In terms of total number of features, the composition 0.5/1.7/0.3 allowed the detection of 1.9, 3.1 and 4.5 times more features than the compositions (0.83/0.83/0.83), (1.1/1.1/0.3), and (1.7/0.5/0.3), respectively. The mean RSD was comprised between 4–10% depending on the solvent composition, with 4% for the condition with more water.

Consequently, for whole fish, the most favorable condition for both intensity and number of features was the one with the highest water content.

*Liver*. In the case of liver, the intensities were generally higher with extraction conditions richer in methanol. This is particularly visible for all hormones. On the other hand, a higher heptane volume induced an increase in the intensity of the amino acids tryptophan and threonine. The signal repeatability with the composition (1.7/0.5/0.3) was good since the mean RSD value was 20%.

The different compositions allowed the detection of an equal number of features with RPLC(ESI+) conditions. On the other hand, with HILIC(ESI-) conditions, more features were detected with a solvent composition richer in methanol. The mean RSD for the number of features was comprised between 6–15% with 7% for the condition with more MeOH.

Consequently, for liver, the most favorable condition considering both intensity and number of features was the one with the highest MeOH content.

*Brain*. For brain extracts, the mean intensities were higher for many of the IS using an extraction mixture richer in MeOH, especially for the amino acids histidine, tryptophan, asparagine and valine. The increase of the MeOH fraction induced an intensity augmentation of 6 amino acids out of 9. In contrast, a higher heptane portion seemed to increase the intensity of the hormones testosterone and progesterone. The mean RSD for intensity was 16% (RPLC) and 24% (HILIC) for the condition rich in MeOH.

The total number of features varied little with a change in solvent the composition (between 1715 and 2118). Consequently, for brain, the solvent composition richest in MeOH was chosen.

*Gills*. Finally, for gills extracts, when considering the results with RPLC conditions, the intensities of the IS were globally slightly higher with the composition (1.1/1.1/0.3) especially for ribose and fucose/rhamnose sugars. With HILIC conditions, the compositions richer in water and the one (1/3, 1/3, 1/3) globally allowed slightly higher intensities. The average RSD values were generally lower with HILIC (between 8.6 and 29) than with RPLC (between 11 and 17%). The total number of features was slightly higher in the conditions (1.1/1.1/0.3). Therefore, the mixture containing equivalent amounts of MeOH and water was considered the best compromise for gills extraction.

### Comparison of the metabolome coverage by the four matrices

**Fig 6:**
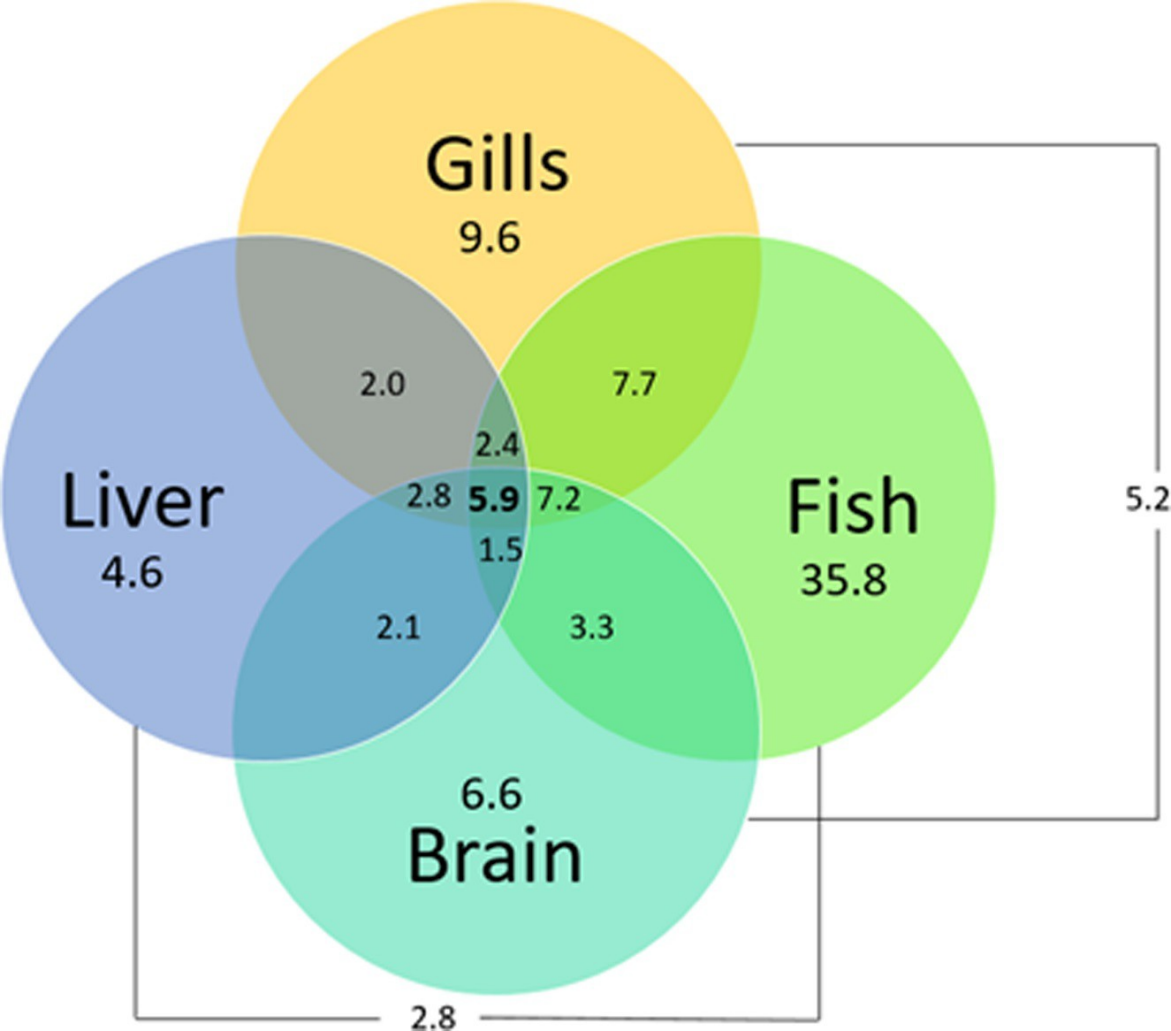
Venn diagram showing specific and overlapping of detected features (m/z, R_T_) achieved with the RPLC(ESI+)/HILIC(ESI-) analyses in brain, gills, liver and fish (expressed in % of total features).

The total number of detected compounds, expressed in percentage of total features, accomplished by using the different LC conditions employed in this study are showed in Fig 6. Unsurprisingly, the total number of compounds detected was lower when the organs compartments were considered individually compared to the whole body.

The total number of features (specific and overlapping) was assigned as followed: whole fish (63.8%) > gills (37.6%) > brain (29.4%) > liver (19.9%). By considering only the two matrices whole fish and gills, a coverage of more than 78% of detectable metabolites was ensured. These results seem consistent since gills serve as an important exchange location for biological reactions with the environment, especially for oxygen exchanges. However, one would have expected to observe more joint compounds with the whole fish than with any other matrix, as it contains all organ compartments. This may be attributed to a dilution of some metabolites present in some organs but absent from others. Consequently, the total number of features in the whole fish is reduced by this dilution effect. Based on these results, it is suggested that when time and/or sample amount are limited for metabolomic analyses, the use of whole fish and gills matrices provide a good compromise to ensure a good coverage of biological information.

**Fig 7:**
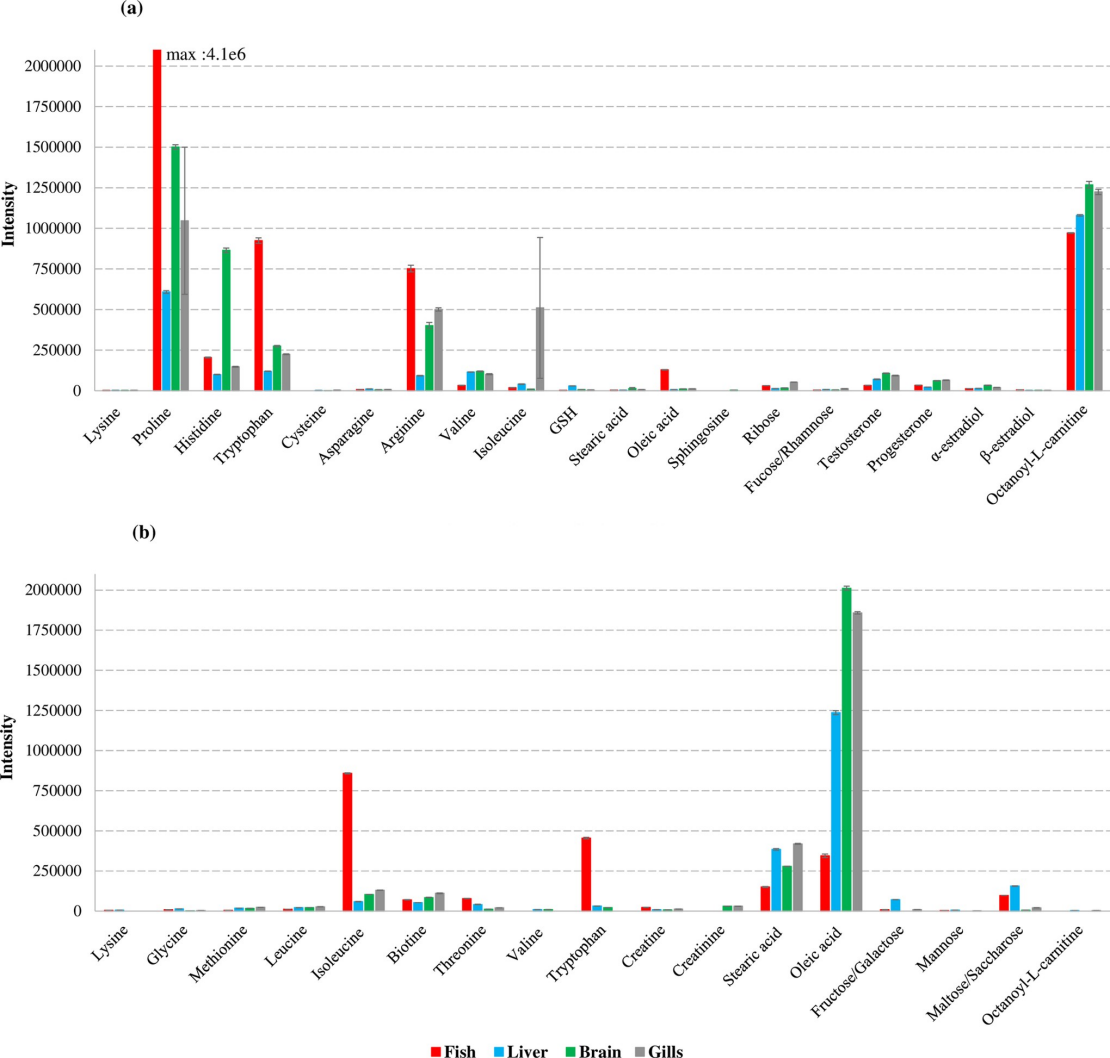
Intensities of targeted metabolites detected in fish, liver, brain and gills, analyzed in (a) RPLC(ESI+) and (b) HILIC(ESI-).

Differences in signal intensity also appeared between the organs compartments and the whole fish (Fig 7). Intensities of proline, tryptophan and arginine were higher in fish while intensities were higher for the hormones testosterone, progesterone and α-estradiol in brain and gills extracts. Better intensities for the sugars ribose, fucose and rhamnose were also observed in gills. On the other hand, sphingosine and cysteine signals were not detected in whole fish; sphingosine was also not detected in liver. Biotin, valine and mannose were not detected in whole fish, while creatine and biotin were not detected in liver.

Analyses of the different compartments are therefore complementary and different approaches can be envisaged between the search for the mode of action of a chemical stressor or the identification of biomarkers that may reflect the disturbance of a more specific function or organ.

## Conclusions

This study describes the development of a reliable method for untargeted metabolomics studies, based on a solid-liquid extraction followed by LC-HRMS analysis, to detect the metabolites contained in brain, gills, liver, and whole fish of the three-spined stickleback. We introduced a single extraction-dual LC-HRMS approach combining RPLC/HILIC-HRMS to measure the maximum number and kind of metabolites in various matrices for non-targeted metabolic profiling. This development allows the study of the amount of information provided by four different compartments in fish with RPLC(ESI+) and HILIC(ESI-). One objective of the present work was to identify the matrix that would detect the most metabolites. Whole fish and gills were found to be the matrices with the highest relative amounts of information.

This study supplies further evidence that extraction, separation and detection methods generate significant differences in metabolomics results. Indeed, it highlights the need to adjust and refine the protocol to obtain the maximum information on the metabolome depending on the tissues or organs considered. Moreover, it provides a useful LC-HRMS-based metabolomics methodology for further study of metabolic perturbations in fish exposed to environmental pollutants. This development would therefore be valuable for the search of potential biomarkers related to environmental exposure and for the assessment of the overall mode of action of pollutants.

## Supporting information

S1 Fig Optimized analytical workflow for the multi-matrices LC-HRMS untargeted metabolomic analysis of three-spined stickleback. (PDF)

S2 Fig Intensities of internal standard metabolites (normalized to the maximal response) analyzed by LC(RPLC)-(ESI+)- or LC(HILIC)-(ESI-)- HRMS after extraction of 50 mg or 25 mg of fish with 2.5 mL or 7 mL of MeOH/H_2_O/heptane. (PDF)

S3 Fig Intensities of internal standard metabolites (normalized to the maximal response) analyzed by LC(RPLC)-(ESI+)- or LC(HILIC)-(ESI-)- HRMS after extraction of 50 mg or 25 mg of liver with 2.5 mL or 7 mL of MeOH/H_2_O/heptane. (PDF)

S4 Fig Intensities of internal standard metabolites (normalized to the maximal response) analyzed by LC(RPLC)-(ESI+)- or LC(HILIC)-(ESI-)- HRMS after extraction of 50 mg or 25 mg of brain with 2.5 mL or 7 mL of MeOH/H_2_O/heptane. (PDF)

S5 Fig Intensities of internal standard metabolites (normalized to the maximal response) analyzed by LC(RPLC)-(ESI+)- or LC(HILIC)-(ESI-)- HRMS after extraction of 50 mg or 25 mg of gills with 2.5 mL or 7 mL of MeOH/H_2_O/heptane. (PDF)

S6 Fig Intensities of internal standard metabolites (normalized to the maximal response) analyzed by LC(RPLC)-(ESI+)- or LC(HILIC)-(ESI-)- HRMS after extraction of 50 mg of fish with 2.5 mL of different proportions of MeOH/H_2_O/heptane. (PDF)

S7 Fig Intensities of internal standard metabolites (normalized to the maximal response) analyzed by LC(RPLC)-(ESI+)- or LC(HILIC)-(ESI-)- HRMS after extraction of 50 mg of liver with 2.5 mL of different proportions of MeOH/H_2_O /heptane. (PDF)

S8 Fig Intensities of internal standard metabolites (normalized to the maximal response) analyzed by LC(RPLC)-(ESI+)- or LC(HILIC)-(ESI-)- HRMS after extraction of 50 mg of brain with 2.5 mL of different proportions of MeOH/H_2_O /heptane. (PDF)

S9 Fig Intensities of internal standard metabolites (normalized to the maximal response) analyzed by LC(RPLC)-(ESI+)- or LC(HILIC)-(ESI-)- HRMS after extraction of 25 mg of gills with 2.5 mL of different proportions of MeOH/H_2_O /heptane. (PDF)

S1 Table Internal standards employed and adducts used during the analytical development. (PDF)
