# Supplementary material for: An optimized LC-HRMS untargeted metabolomics workflow for multi-matrices investigations in the three-spined stickleback
Source: PLoS One. 2021 Nov 29;16(11):e0260354. doi: 10.1371/journal.pone.0260354 (PMC8629232; doi:10.1371/journal.pone.0260354)

Figure S1 : Optimized analytical workflow for the multi-matrices LC-HRMS untargeted metabolomic analysis of three-spined stickleback

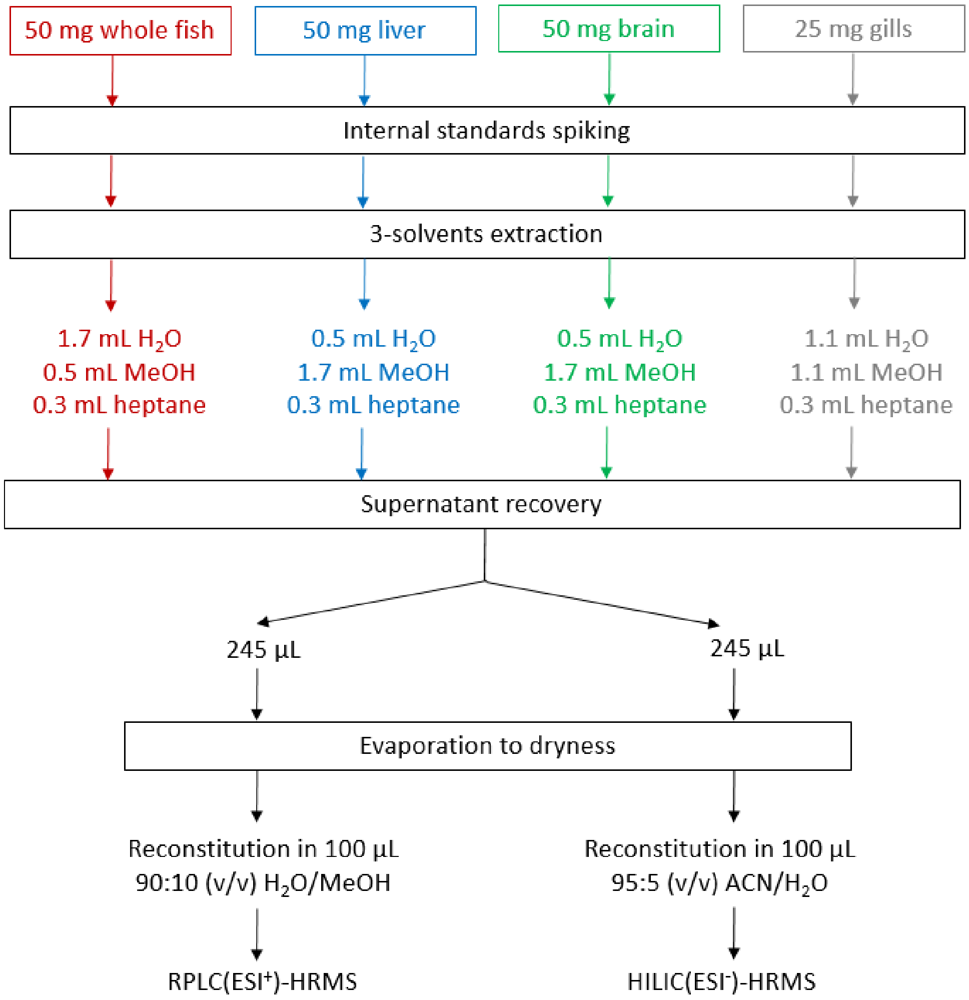

Supplement: S1 Fig — (PDF) [file pone.0260354.s001.pdf]
