# Supplementary material for: An optimized LC-HRMS untargeted metabolomics workflow for multi-matrices investigations in the three-spined stickleback
Source: PLoS One. 2021 Nov 29;16(11):e0260354. doi: 10.1371/journal.pone.0260354 (PMC8629232; doi:10.1371/journal.pone.0260354)

Figure S9. Normalized intensities of internal metabolites analyzed by LC(RPLC)-(ESI+)- or LC(HILIC)-(ESI-)- HRMS after extraction of 25 mg of gills with 2.5 mL of different proportions of MeOH/H_2O/heptane.

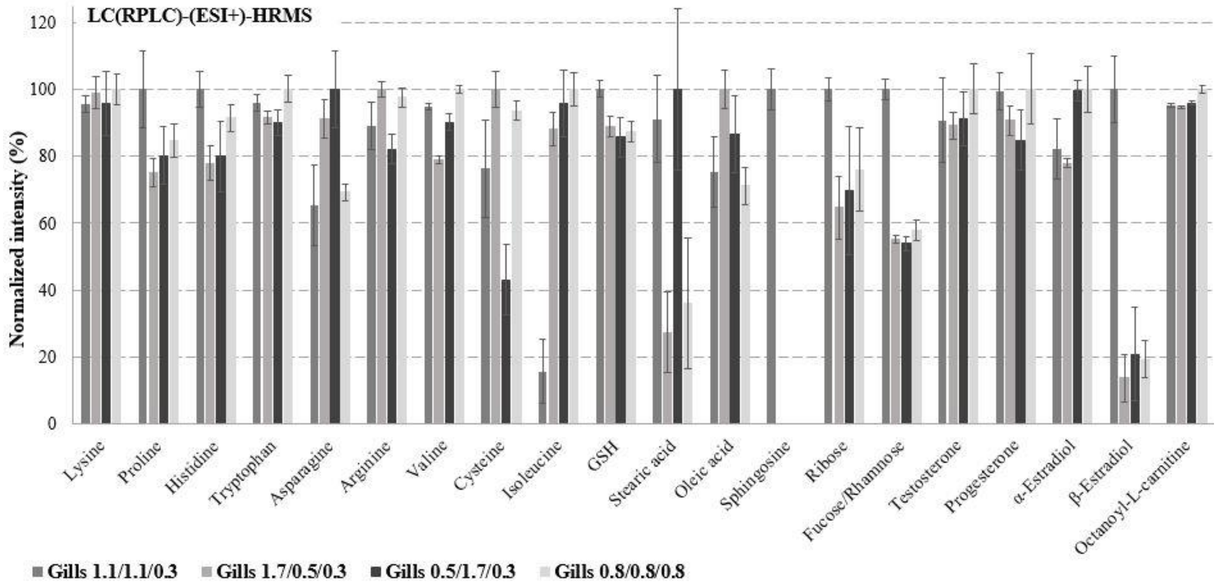

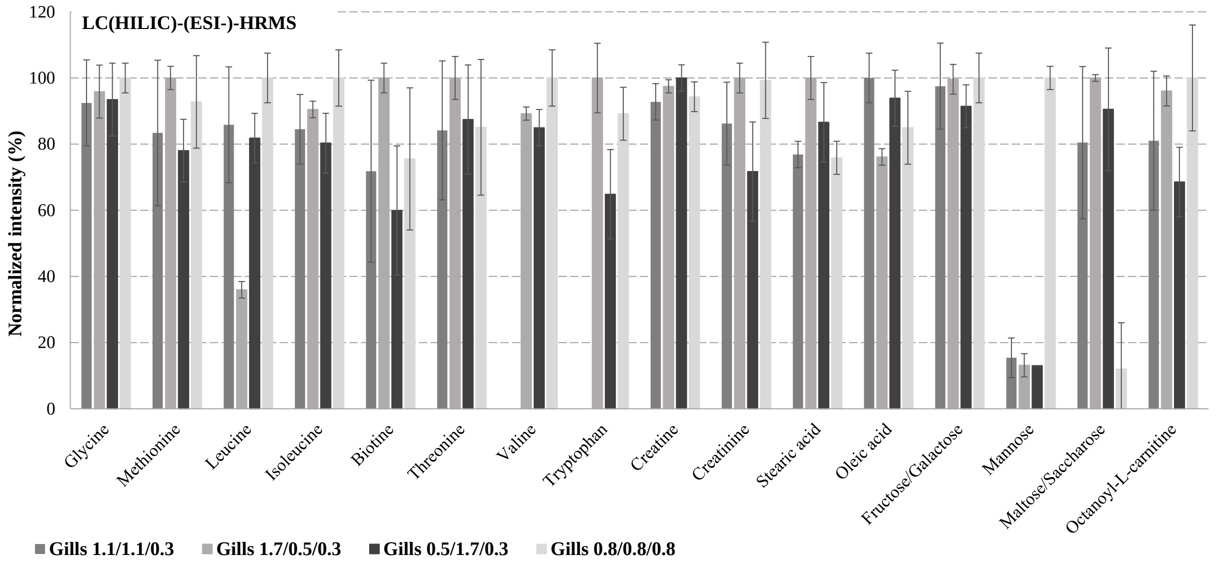

Supplement: S9 Fig — (PDF) [file pone.0260354.s009.pdf]
