# Supplementary material for: An optimized LC-HRMS untargeted metabolomics workflow for multi-matrices investigations in the three-spined stickleback
Source: PLoS One. 2021 Nov 29;16(11):e0260354. doi: 10.1371/journal.pone.0260354 (PMC8629232; doi:10.1371/journal.pone.0260354)
Supplement: S1 Table — (PDF) [file pone.0260354.s010.pdf]

Table S1. Internal standards employed and adducts used during the analytical development

Compounds	Chemical group	Supplier	Purity (%)	Formula	Adduct detected mass (Da)	Chromatographic condition
Stearic acid	Fatty acid	Sigma-aldrich	> 95.0	$C_{18}H_{36}O_2$	$[M+H]^+$ 285.2788<br>$[M-H]^-$ 283.2631	RPLC<br>HILIC
Sphingosine	Sphingolipid	Sigma-aldrich	> 95.0	$C_{19}H_{37}NO_2$	$[M+H]^+$ 312.2897	RPLC
Oleic acid	Fatty acid	Sigma-aldrich	> 95.0	$C_{18}H_{34}O_2$	$[M+H]^+$ 283.2632<br>$[M-H]^-$ 281.2475	RPLC<br>HILIC
Palmitic acid	Fatty acid	Sigma-aldrich	98.0	$D_{31}C_{16}O_2H$	$[M+H]^+$ 288.4421<br>$[M+Na]^+$ 310.4240<br>$[M-H]^-$ 286.4264<br>$[M-H]^-$ 286.4264	RPLC<br>RPLC<br>RPLC<br>HILIC
Cholesterol	Lipid sterol	Sigma-aldrich	99.0	$^{13}C_3C_{24}H_{46}O$	$[M-H]^-$ 388.3566	HILIC
Valine	Amino acid	Sigma-aldrich	98.0	$^{15}NC_5H_{11}O_2$	$[M+H]^+$ 119.0833<br>$[M-H]^-$ 117.0676<br>$[M+H]^+$ 119.0833<br>$[M-H]^-$ 117.0676	RPLC<br>RPLC<br>HILIC<br>HILIC
Glutamine	Amino acid	Sigma-aldrich	98.0	$^{15}NC_5H_{10}NO_3$	$[M+H]^+$ 148.0735<br>$[M-H]^-$ 146.0578<br>$[M-H]^-$ 146.0578	RPLC<br>RPLC<br>HILIC
Glycine	Amino acid	Sigma-aldrich	99.0	$^{13}CNCO_2H_5$	$[M+H]^+$ 77.0427<br>$[M-H]^-$ 75.0270<br>$[M+H]^+$ 77.0427<br>$[M-H]^-$ 75.0270	RPLC<br>RPLC<br>HILIC<br>HILIC
Lysine	Amino acid	Sigma-aldrich	98.0	$D_4N_2C_6O_2H_{10}$	$[M+H]^+$ 151.1379	RPLC
Methionine	Amino acid	Sigma-aldrich	98.0	$C_5D_3H_8NO_2S$	$[M+H]^+$ 153.0772<br>$[M-H]^-$ 151.0615<br>$[M+H]^+$ 153.0772	RPLC<br>RPLC<br>HILIC

					$[M-H]^-$ 151.0615	HILIC
Leucine	Amino acid	Sigma-aldrich	98.0	$^{13}C_6H_{13}(^{15}N)O_2$	$[M+H]^+$ 139.1191<br>$[M-H]^-$ 137.1034<br>$[M+H]^+$ 139.1191<br>$[M-H]^-$ 137.1034	RPLC<br>RPLC<br>HILIC<br>HILIC
Tryptophan	Amino acid	-	> 95.0	$C_{11}H_{12}N_2O_2$	$[M+H]^+$ 205.0972<br>$[M-H]^-$ 203.0815	RPLC<br>HILIC
Asparagine	Amino acid	Sigma-aldrich	98.0	$C_4H_8N_2O_3$	$[M+H]^+$ 133.0608	RPLC
Histidine	Amino acid	Sigma-aldrich	> 95.0	$C_6H_9N_3O_2$	$[M+H]^+$ 156.0768	RPLC
Threonine	Amino acid	Fluka	99.0	$C_4H_9NO_3$	$[M+H]^+$ 120.0655<br>$[M-H]^-$ 118.0499	RPLC<br>HILIC
Arginine	Amino acid	Fluka	99.5	$C_6H_{14}N_4O_2$	$[M+H]^+$ 175.1190	RPLC
Cysteine	Amino acid	Sigma-aldrich	97.0	$C_3H_7NO_2S$	$[M+H]^+$ 122.0270	RPLC
Isoleucine	Amino acid	Sigma-aldrich	98.0	$C_6H_{13}NO_2$	$[M+H]^+$ 132.1019<br>$[M-H]^-$ 130.0862	RPLC<br>HILIC
Proline	Amino acid	-	> 95.0	$C_5H_9NO_2$	$[M+H]^+$ 116.0706	RPLC
Aspartic acid	Amino acid	Sigma-aldrich	98.0	$^{15}NC_4O_4H_7$	$[M+H]^+$ 135.0418<br>$[M-H]^-$ 133.0262	RPLC<br>RPLC
Creatine	Derivative amino acid	ChemService	> 95.0	$C_4H_9N_3O_2$	$[M+H]^+$ 132.0768<br>$[M-H]^-$ 130.0611	RPLC<br>HILIC
Creatinine	Derivative amino acid	Sigma-aldrich	> 95.0	$C_4H_7N_3O$	$[M+H]^+$ 114.0662<br>$[M-H]^-$ 112.0505	RPLC<br>HILIC
Lactic acid	Organic acid	Sigma-aldrich	98.0	$^{13}CC_2H_6O_3$	$[M+H]^+$ 91.0345<br>$[M-H]^-$ 89.0189	HILIC<br>HILIC
Taurocholic acid	Biliar acid	Clinisciences	99.0	$C_{26}H_{39}D_5NO_7S$	$[M+H]^+$520.3225<br>$[M-H]^-$ 518.3069<br>$[M+H]^+$ 520.3225<br>$[M-H]^-$ 518.3069	RPLC<br>RPLC<br>HILIC<br>HILIC

Octanoyl-L-carnitine	Peptide	Sigma-aldrich	97.0	$C_{15}D_3H_{26}NO_4$	$[M+H]^+$ 291.2358<br>$[M+Na]^+$ 313.2177<br>$[M-H]^-$ 289.2201<br>$[M+H]^+$ 291.2358<br>$[M-H]^-$ 289.2201	RPLC<br>RPLC<br>RPLC<br>HILIC<br>HILIC
GSH	Peptide	Sigma-aldrich	98.0	$C_{10}H_{17}N_3O_6S$	$[M+H]^+$ 308.0911	RPLC
Biotine	Vitamin	Sigma-aldrich	98.0	$C_{10}D_2H_{14}N_2O_3S$	$[M+H]^+$ 247.1080<br>$[M-H]^-$ 245.0923	HILIC<br>HILIC
α-Estradiol	Hormone	Sigma-Aldrich	99.9	$C_{18}H_{24}O_2$	$[M+H]^+$ 273.1849<br>$[M-H]^-$ 271.1693	RPLC<br>RPLC
β-Estradiol	Hormone	CDN isotopes	99.5	$C_{18}H_{22}D_2O_2$	$[M+H]^+$ 275.1975<br>$[M-H]^-$ 273.1818<br>$[M+H]^+$ 275.1975<br>$[M-H]^-$ 273.1818	RPLC<br>RPLC<br>HILIC<br>HILIC
Testosterone	Hormone	CDN isotopes	99.7	$C_{19}H_{26}D_2O_2$	$[M+H]^+$ 291.2288<br>$[M+H]^+$ 291.2288<br>$[M-H]^-$ 289.2131	RPLC<br>HILIC<br>HILIC
Progesterone	Hormone	CDN isotopes	98.5	$C_{21}H_{21}D_9O_2$	$[M+H]^+$ 324.2883<br>$[M+H]^+$ 324.2883	RPLC<br>HILIC
Estrone	Hormone	CDN isotopes	99.2	$C_{18}H_{20}D_2O_2$	$[M+H]^+$ 273.3873<br>$[M-H]^-$ 271.3716<br>$[M+H]^+$ 273.3873<br>$[M-H]^-$ 271.3716	RPLC<br>RPLC<br>HILIC<br>HILIC
Glucose	Sugar	Sigma-aldrich	99.0	$^{13}C_6H_{12}O_6$	$[M+H]^+$ 187.0908<br>$[M-H]^-$ 185.0751<br>$[M+H]^+$ 187.0908<br>$[M-H_20-H]^-$ 167.0646<br>$[M-H]^-$ 185.0751	RPLC<br>RPLC<br>HILIC<br>HILIC<br>HILIC
Mannose	Sugar	Sigma-aldrich	99.0	$^{13}CC_5H_{12}O_6$	$[M+H]^+$ 182.0740<br>$[M-H]^-$ 180.0584<br>$[M+H]^+$ 182.0740<br>$[M-H_2O-H]^-$ 162.478	RPLC<br>RPLC<br>HILIC<br>HILIC

					$[M-H]^-$ 180.0584	HILIC
Fucose	Sugar	Sigma-aldrich	98.0	$C_6H_{12}O_5$	$[M+H]^+$ 165.0758<br>$[M+Na]^+$ 187.0577<br>$[M+NH_4]^+$ 182.1023	RPLC<br>RPLC<br>RPLC
Rhamnose	Sugar	-	> 95.0	$C_6H_{12}O_5$	$[M+H]^+$ 165.0758<br>$[M+Na]^+$ 187.0577<br>$[M+NH_4]^+$ 182.1023	RPLC<br>RPLC<br>RPLC
Fructose	Sugar	Sigma-aldrich	99.0	$C_6H_{12}O_6$	$[M+H]^+$ 181.0707<br>$[M-H_2O-H]^-$ 161.0445	RPLC<br>HILIC
Galactose	Sugar	Sigma-aldrich	98.0	$C_6H_{12}O_6$	$[M+H]^+$ 181.0707<br>$[M-H_2O-H]^-$ 161.0445	RPLC<br>HILIC
Saccharose	Sugar	Merck	> 95.0	$C_{12}H_{22}O_{11}$	$[M+H]^+$ 343.1235<br>$[M-H_2O-H]^-$ 323.0973	RPLC<br>HILIC
Maltose	Sugar	Sigma-aldrich	99.0	$C_{12}H_{22}O_{11}$	$[M+H]^+$ 343.1235<br>$[M-H_2O-H]^-$ 323.0973	RPLC<br>HILIC
Ribose	Sugar	Sigma-aldrich	98.0	$C_5H_{10}O_5$	$[M+H]^+$ 151.0601	RPLC
